# Supplementary material for: Knowledge about Cervical Cancer and Awareness of Human Papillomavirus (HPV) and HPV Vaccine among Female Students from Serbia
Source: Medicina (Kaunas). 2020 Aug 13;56(8):406. doi: 10.3390/medicina56080406 (PMC7466248; doi:10.3390/medicina56080406)
Supplement: Supplementary file 1 [file medicina-56-00406-s001.pdf]

Example of the questionnaire

Section 1:

1. Your age
2. College attending: 1)**Medical** 2)**Other**
3. Mother's level of education: 1)**Low** 2)**Middle** 3)**High**
4. Father's level of education: 1)**Low** 2)**Middle** 3)**High**
5. Place of residence: 1)**Urban area** 2)**Rural area**
6. Type of high school finished: 1)**Gymnasium** 2)**Other non-medical high school** 3)**Medical high school**
7. Financial status/income: 1)**Low** 2)**Middle** 3)**High**
8. Relationship status: 1)**Single** 2)**In relationship** 3)**Married** 4)**Divorced** 5)**Widow**
9. Ever had sexual intercourse: 1)**Yes** 2)**No**
10. Number of sexual partners (if ever had sexual intercourse): 1)**1** 2)**2-4** 3)**5+**

Section 2:

Item	Offered answers	Points for correct answer
1. Sexual onset before age of 16	Risk factor*/Protective factor /Don't know	2
2. Numerous labors	Risk factor*/Protective factor /Don't know	2
3. High number of sexual partners	Risk factor*/Protective factor /Don't know	2
4. Use of oral contraceptives	Risk factor*/Protective factor /Don't know	2
5. Use of condom	Risk factor*/Protective factor /Don't know	2
6. HPV infection	Risk factor*/Protective factor /Don't know	2
7. Other STD's	Risk factor*/Protective factor /Don't know	
8. Weaken immune system	Risk factor*/Protective factor /Don't know	2
9. Smoking	Risk factor*/Protective factor /Don't know	2
10. Regular gynecological checkups /screening	Risk factor/Protective factor* /Don't know	2
11. Family history of CC	Risk factor*/Protective factor /Don't know	1
12. STD's in sexual partner	Risk factor*/Protective factor /Don't know	1
13. Obesity	Risk factor*/Protective factor /Don't know	1
14. Healthy diet	Risk factor/Protective factor* /Don't know	1
15. Physical activity	Risk factor/Protective factor* /Don't know	1
16. Screening programs enable early detection of cervical abnormalities	True*/Falls/Don't know	2
17. The Pap test checks for changes in the cells of the cervix	True*/Falls/Don't know	2
18. CC is highly treatable when caught early	True*/Falls/Don't know	2

1. Have you heard about HPV? 1)**Yes** 2)**No**

1. Have you heard about HPV vaccine? 1)**Yes** 2)**No**
2. When is the best time to get the HPV vaccine? 1)**Before becoming sexually active*** 2)**Just after first sexual intercourse** 3)**It doesn't matter when** 4)**Don't know**
3. Is HPV vaccine available in Serbia? 1)**Yes*** 2)**No** 3)**Don't know**

*correct answer

Section 3

Source of your knowledge and information about cervical cancer is:

1. Physicians[a]
2. Nurses[a]
3. School teachers[a]
4. Lectures /speeches[a]
5. Pamphlet/brochure[a]
6. Medical literature[a]
7. Parents/guardian[b]
8. Other relatives[b]
9. Partners/boyfriends[b]
10. Friends[b]
11. Newspapers/magazines[c]
12. TV/radio advertisements[c]
13. The Internet[c]
14. I don't have any knowledge

[a] personal contact; [b] organized health education; [c] media
